# Gentian violet induces wtp53 transactivation in cancer cells

**DOI:** 10.3892/ijo.2014.2304

**Published:** 2014-02-14

**Authors:** ALESSIA GARUFI, VALERIO D’ORAZI, JACK L. ARBISER, GABRIELLA D’ORAZI

**Affiliations:** 1 Department of Experimental Oncology, Regina Elena National Cancer Institute, 00159 Rome;; 2 Department of Surgical Sciences, Sapienza University, 00161 Rome, Italy;; 3 Department of Dermatology and Winship Cancer Institute, Emory University School of Medicine, Atlanta Veterans Administration Medical Center, Atlanta, GA, USA;; 4 Department of Medical, Oral and Biotechnological Sciences, University ‘G. d’Annunzio’, 66013 Chieti, Italy

**Keywords:** p53 transcriptional activity, gentian violet, cancer therapy, DNA binding, gene expression, NADPH oxidase 1

## Abstract

Recent studies suggest that gentian violet (GV) may have anticancer activity by inhibiting for instance NADPH oxidases (Nox genes) whose overexpression is linked to tumor progression. Nox1 overexpression has been shown to inhibit transcriptional activity of the oncosuppressor p53, impairing tumor cell response to anticancer drugs. The tumor suppressor p53 is a transcription factor that, upon cellular stress, is activated to induce target genes involved in tumor cell growth inhibition and apoptosis. Thus, its activation is important for efficient tumor eradication. In this study, we examined the effect of GV on wild-type (wt) p53 activity in cancer cells. We found that GV was able to overcome the inhibitory effect of the NADPH oxidase Nox1 on p53 transcriptional activity. For the first time we show that GV was able to directly induce p53/DNA binding and transcriptional activity. *In vitro*, GV markedly induced cancer cell death and apoptotic marker PARP cleavage in wtp53-carrying cells. GV-induced cell death was partly inhibited in cells deprived of p53, suggesting that the anticancer activity of GV may partly depend on p53 activation. GV is US Food and Drug Administration approved for human use and may, therefore, have therapeutic potential in the management of cancer through p53 activation.

## Introduction

The oncosuppressor p53 plays a critical role in cancer cell growth inhibition and apoptosis in response to a variety of stress signals, including DNA damage, hypoxia and aberrant proliferation signals such as oncogene activation (1). Most of the p53 oncosuppressor activities, such as cell cycle arrest, apoptosis, or senescence, are achieved through the control of p53-mediated gene transcription (2). The activation of the p53 pathway has been implicated in an individual’s ability to suppress tumor formation and to respond to many types of cancer therapies (3). Therefore, p53 is the most inactivated oncosuppressor in human tumors mainly by gene mutations or deregulation of protein activity (4). In this regard, we previously showed that p53 apoptotic activity may be inhibited by overexpression of NADPH oxidase 1 (Nox1) that interferes with p53 binding to apoptotic gene promoters by affecting p53 post-translational modifications (5,6). NADPH oxidases are a family of enzymes that regulate redox-sensitive signalling pathways involved in cancer development and progression and are often overexpressed in different types of human cancers favouring tumor progression and angiogenesis (7,8).

Cationic triphenylmethane pharmacophore (TPM) gentian violet (GV) has a long history of use as antifungal and antibacterial agent (9) and is also exploited in other research fields (10). Of note, GV has been shown to have potent anticancer and anti-angiogenic activities in mice and human (11,12) and to have an effect against melanoma metastases (13), although the anticancer molecular mechanisms are not fully understood. GV was originally shown to have oxidation-reduction potential (14) with likely free radical formation. Recent studies showed that GV may have potent inhibitory activity against NADPH oxidase Nox2 and Nox4 (15) and to induce cell death by disruption of the mitochondrial system (16). GV enhances the cytotoxic activity of thiostrepton (TS), a thiazole antibiotic that inhibits the expression of oncogenic transcription factor FOXM1, required for cell cycle progression and resistance to oncogene-induced oxidative stress (17). The GV and TS combinatorial approach may thus be particularly useful in treating tumors with aberrant mitochondrial oxidant production.

The success of GV in inhibiting Nox activity and to induce anticancer effects prompted us to examine the potential effect of this agent on wild-type (wt) p53 activity, which has not been addressed yet. We found that GV counteracted the Nox1 inhibitory effect toward p53 transcriptional activity, restoring p53 transactivation. Noteworthy, GV was able to directly induce p53 oncosuppressor activity by stimulating p53/DNA binding and transactivation activities. Therefore, GV anticancer activity may depend also on p53 activation.

## Materials and methods

### Cell culture and reagents

Human lung cancer H1299 (p53 null), colon cancer RKO (carrying wt-p53), RKO stable interfered for p53 function (RKO-sip53) (18) (a kind gift from S. Soddu, Regina Elena National Cancer Institute, Rome, Italy), colon cancer HCT116 (wtp53), HCT116-p53^−/−^ (a kind gift from B. Vogelstein, Johns Hopkins University, Baltimore, MD), glioblastoma ADF (wtp53) (19,20) cell lines were routinely maintained in RPMI-1640 (Life-Technology-Invitrogen) medium containing 10% heat-inactivated fetal bovine serum (FBS), 100 U/ml penicillin/streptomycin, and glutamine, in 5% CO_2_ humidified incubator at 37°C.

The following reagents were used: gentian violet (GV) stored at 1 mM in DMSO was used at 0.2, 0.5, 1, and 2 *μ*M; p53 inhibitor pifithryn-α (PFT) (21) (Enzo Life Sciences, Lausen, Switzerland) was used at 30 *μ*M; chemotherapeutic agent adriamycin (ADR) stored at 2 *μ*g/*μ*l in PBS was used at 2 *μ*g/ml. PBS and DMSO solvents were used as control.

### RNA isolation and reverse transcription (RT)-PCR analysis

Cells were harvested in TRIzol Reagent and total RNA was isolated following the manufacturer’s instructions (Invitrogen). The first-strand cDNA was synthesized from 2 *μ*g of total RNA with MuLV reverse transcriptase kit (Applied Biosystems). Semi-quantitative reverse-transcribed (RT)-PCR was carried out by using Hot-Master Taq polymerase (Eppendorf) with 2 *μ*l cDNA reaction and genes specific oligonucleotides under conditions of linear amplification. PCR products were run on a 2% agarose gel and visualized with ethidium bromide. The housekeeping β-actin gene, used as internal standard, was amplified from the same cDNA reaction mixture. Densitometric analysis was applied to quantify mRNA levels compared to control gene expression.

### Viability assay

Exponentially proliferating cells were exposed to different concentrations of GV for 24 h. Both floating and adherent cells were collected and counted in hemocytometer after addition of trypan blue. The percentage of dead cells (i.e. blue/total cells) was determined by scoring 100 cells per chamber for three times. At least three independent experiments were performed and cell numbers were determined in triplicates.

### Western blotting

Total cell extracts were prepared by incubation in lysis buffer containing 50 mmol/l Tris-HCl, pH 7.5, 150 mmol/l NaCl, 150 mmol/l KCl, 1 mmol/l dithiothreitol, 5 mmol/l EDTA, pH 8.0, 1% Nonidet P-40 plus a mix of protease and phosphatase inhibitors (Roche Diagnostic) and resolved by SDS-polyacrylamide gel electrophoresis. Proteins were transferred to a polyvinylidene difluoride membrane (PVDF, Millipore) and incubated with the primary antibodies followed by an anti-immunoglobulin-G-horseradish peroxidase antibody (Bio-Rad). Specific proteins were detected by enhanced chemiluminescence (ECL) (Amersham). Immunoblotting was performed with: mouse monoclonal anti-Poly (ADP-ribose) polymerase (PARP) (cleaved form, BD Pharmingen), rabbit polyclonal anti-p53 (FL393) mouse monoclonal anti-p53 (DO1) (both from Santa Cruz Biotechnology), monoclonal anti-GFP (Roche Diagnostic), polyclonal anti-Bax (N20, Santa Cruz Biotechnology), monoclonal anti-phospho-Histone H2A.X (Ser139) (Millipore) (a kind gift from S. Soddu) and monoclonal anti-β-actin (Calbiochem) antibodies.

### Transfection and transactivation assay

For the assay, H1299 cells, plated at subconfluence in 60-mm Petri dishes, were transiently co-transfected using the N,N-bis-(2- hydroxyethyl)-2-amino-ethanesulphonic acid-buffered saline (BBS) version of the calcium phosphate procedure (22) with the luciferase reporter gene driven by the p53-dependent natural Noxa-luc (kindly provided by T. Taniguchi, University of Tokyo, Japan) promoter, wtp53 (0.5 *μ*g/sample) and Nox1-GFP (4 *μ*g/sample) (kindly provided by M. Bignami, National Institute of Health, ISS, Rome, Italy) expression vectors. Twenty-four hours after transfection cells were treated with GV (1 *μ*M) for further 24 h. Transfection efficiency was normalized with the use of a co-transfected β-galactosidase plasmid. Luciferase activity was assayed on whole cell extract and the luciferase values were normalized to β-galactosidase activity and protein content.

### Chromatin immunoprecipitation (ChIP) assay

ChIP analysis was carried out essentially as previously described (23). Briefly, cells were crosslinked with 1% formaldehyde for 10 min at room temperature and then inactivated by the addition of 125 mM glycine. Chromatin extracts containing DNA fragments with an average size of 500 bp were incubated overnight at 4°C with milk, shaking using polyclonal anti-p53 antibody (FL393, Santa Cruz Biotechnology). Before use, protein G (Pierce) was blocked with 1 *μ*g/*μ*l sheared herring sperm DNA and 1 *μ*g/*μ*l BSA for 3 h at 4°C and then incubated with chromatin and antibody for 2 h at 4°C. PCR was performed with Hot-Master Taq (Eppendorf) using 2 *μ*l of immunoprecipitated DNA and promoter-specific primers spanning p53 binding sites. Immunoprecipitation with non-specific immunoglobulins (IgG, Santa Cruz Biotechnology) was performed as negative controls. PCR products were run on a 2% agarose gel and visualized with ethidium bromide.

### Statistical analysis

Each experiment, unless otherwise specified, was performed at least three times, and data are presented as the mean ± SD. Statistical significance was determined using Student’s t-test. A P-value of ≤0.05 was considered statistically significant.

## Results

### GV counteracts the Nox1 inhibitory effect on p53 transcriptional activity

**Figure 1.**
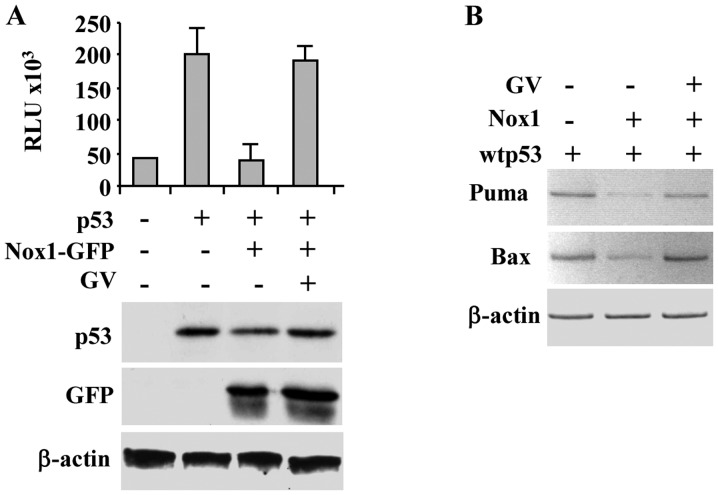
Gentian violet (GV) counteracts the Nox1 inhibitory effect on p53 transcriptional activity. (A) H1299 cells were co-transfected with Noxa-luc (1 *μ*g) reporter and wtp53 (0.5 *μ*g/sample) alone or in combination with GFP-Nox1 (4 *μ*g/sample) expression vectors. Twenty-four hours after transfection GV (1 *μ*M) was added for 24 h before luciferase activity was assayed. The shown data represent the mean ± SD from three independent experiments performed in duplicate. ^*^P<0.001. RLU, relative luciferase unit. (B) Semi-quantitative RT-PCR analyses of p53 target genes in H1299 cells transfected and treated with GV as in (A). β-actin was used as internal control.

Nox1 overexpression in cancer cells has been recently shown to inhibit p53 apoptotic transcriptional activity either after p53 overexpression or after drug-induced p53 activation (6). On the other hand, GV has been reported to efficiently inhibit Nox activity and block tumor growth in mice (15). Therefore, we evaluated whether GV could re-establish wtp53 transcriptional activity in the presence of Nox1 overexpression. To test this hypothesis, luciferase assay was performed in p53-null H1299 cells co-transfected with wtp53 and Nox1-GFP expression vectors and the report vector containing the luciferase gene under the control of p53 target Noxa gene promoter (Noxa-luc). As shown in Fig. 1A, Nox1 repressed the p53-induced Noxa-luciferase reporter activity, as previously reported (6), while GV treatment efficiently restored wtp53 transcriptional activity. Western immunoblotting of the overexpressed proteins is shown (Fig. 1A, lower panel). The positive effect of GV on rescue of p53 transcriptional activity despite Nox1 overexpression was confirmed by *in vivo* RT-PCR analysis whereas the p53-induced target gene transcription (i.e., Puma and Bax) was inhibited by Nox1 co-transfection and restored by concomitant GV treatment (Fig. 1B). These results support the hypothesis that GV inhibits Nox1 activity restoring wtp53 transcriptional activity.

### GV induces γH2AX phosphorylation and p53 protein stabilization

**Figure 2.**
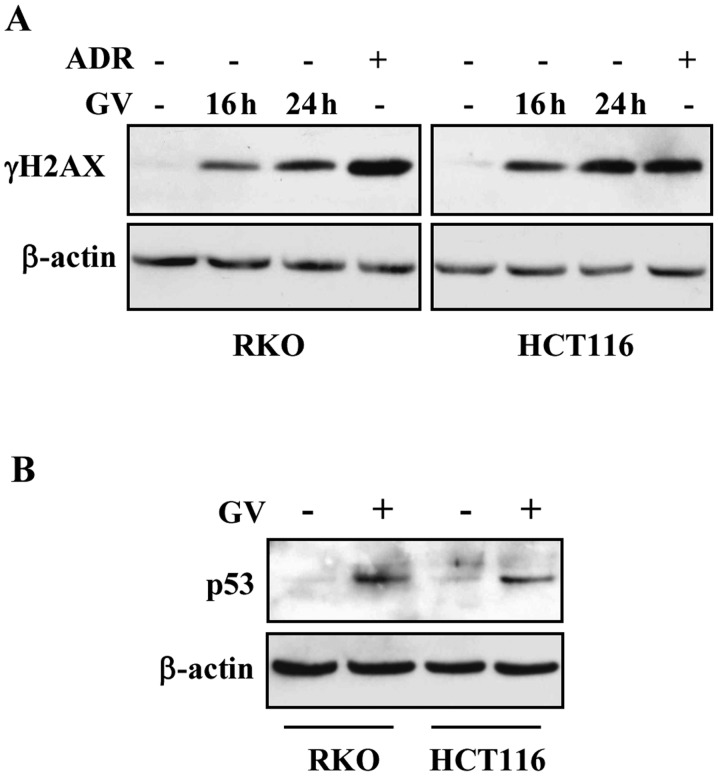
Gentian violet (GV) induces DNA damage with p53 stabilization. (A) RKO and HCT116 cells were treated with GV (1 *μ*M) for 16 and 24 h. Western immunoblotting was performed on equal amount of total cell extracts to detect phospho-Histone H2A.X (γH2AX) levels. Adriamycin (ADR) (2 *μ*g/ml) was used as control of DNA damage. Anti-β-actin was shown as protein loading control. (B) RKO and HCT116 cells were treated with GV (1 *μ*M) 24 h. Western immunoblotting was performed on equal amount of total cell extracts to detect p53 levels. Anti-β-actin was used as protein loading control.

Next, we evaluated whether GV treatment might induce histone H2AX phosphorylation. The phosphorylation of the subtype of histone H2A, called H2AX, in the position of Ser139 producing γH2AX, occurs in response to formation of double strand brakes (DSB) and is an early sign of replication stalling. In general, analysis of γH2AX expression can be used to detect the genotoxic effect of different anticancer agents (24). Herein we found that GV treatment produced γH2AX expression in both RKO and HCT116 cells (Fig. 2A). As a positive control we treated cells with the chemotherapeutic adriamycin (ADR) that indeed efficiently phosphorylated histone H2AX (Fig. 2A). Next, western immunoblotting showed that GV treatment induced p53 protein stabilization (Fig. 2B), suggesting that the effect induced on DNA by GV may be responsible for p53 activation.

### GV induces p53/DNA binding and transcriptional activity

**Figure 3.**
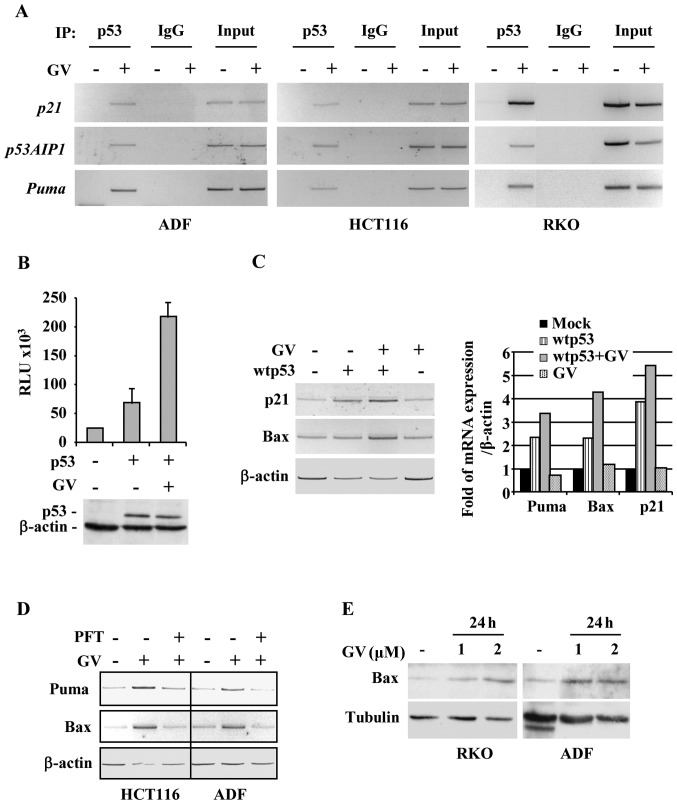
Gentian violet (GV) induces p53/DNA binding and transcriptional activity. (A) ADF, HCT116 and RKO cells (4×10^6^) were plated in 150-mm dish and the day after treated with GV (1 *μ*M) for 16 h before assayed for chromatin immunoprecipitation (ChIP) analysis with anti-p53 (FL393) antibodies. PCR analyses were performed on the immunoprecipitated DNA samples using primers specific for wtp53 target gene promoters (p21, Puma, p53AIP1). A sample representing linear amplification of the total chromatin (Input) was included as control. Additional controls included immunoprecipitation performed with non-specific immunogloblulins (IgG). (B) H1299 cells were co-transfected with Noxa-luc (1 *μ*g) reporter and wtp53 (0.5 *μ*g/sample) expression vector. Twenty-four hours after transfection GV (1 *μ*M) was added for 24 h before luciferase activity was assayed. The shown data represent the mean ± SD from three independent experiments performed in duplicate. Relative luciferase unit. (C, left panel) Semi-quantitative RT-PCR analyses of p53 target genes in H1299 cells transfected and treated with GV as in (B). β-actin was used as internal control. (C, right panel) Gene expression was measured by densitometric analyses and plotted as fold of mRNA expression over control (Mock), normalized to β-actin levels, ± SD. (D) HCT116 and ADF cells were plated at subconfluence in 60-mm Petri dish and the day after treated with GV (1 *μ*M) in the presence or absence of pifithryin-α (PFT-α) (30 *μ*M), for 24 h. P53 target gene expression was detected by RT-PCR analyses. β-actin was used as internal control. (E) RKO and ADF cells were treated with GV (1 and 2 *μ*M) for 24 h. Western immuno blotting was performed on equal amount of total cell extracts to detect Bax levels. Anti-β-actin was used as protein loading control.

We then explored whether GV could directly induce p53 transcriptional activity. To this aim, *in vivo* p53-DNA binding activity was analysed by chromatin immunoprecipitation (ChIP) technique. Cells untreated or treated with GV were cross-linked with formaldehyde, p53 was immunoprecipitated and co-precipitated p53-bound elements were analysed by PCR. The results show that p53 was efficiently recruited onto canonical target promoters, such as p21, p53AIP1 and Puma, after GV treatment in all cancer cell lines analysed (Fig. 3A). Then, H1299 cells were co-transfected with Noxa-luc reporter plasmid and wtp53 expression vector and treated with GV. As shown in Fig. 3B, GV remarkably increased wtp53 transcriptional activity. In agreement with the luciferase results, the *in vivo* wtp53-induced p21 and Bax gene transcription was further increased after GV treatment, as also indicated by densitometric analyses (Fig. 3C). The role of p53 in transcriptional activation of target genes was confirmed by the use of pifithryn-α (PFT-α), an inhibitor of p53 transactivation function (21). As shown in Fig. 3D, the GV-induced p53 target gene transcription was efficiently impaired by PFT-α co-treatment. Then, the effect of GV on Bax protein expression was evaluated by western immunoblotting. As shown in Fig. 3E, GV caused an increase of Bax protein levels in RKO and ADF cells. These data demonstrate that GV is able to directly induce p53/DNA binding and transactivation activities.

### p53 is involved in GV-induced cancer cell death

**Figure 4.**
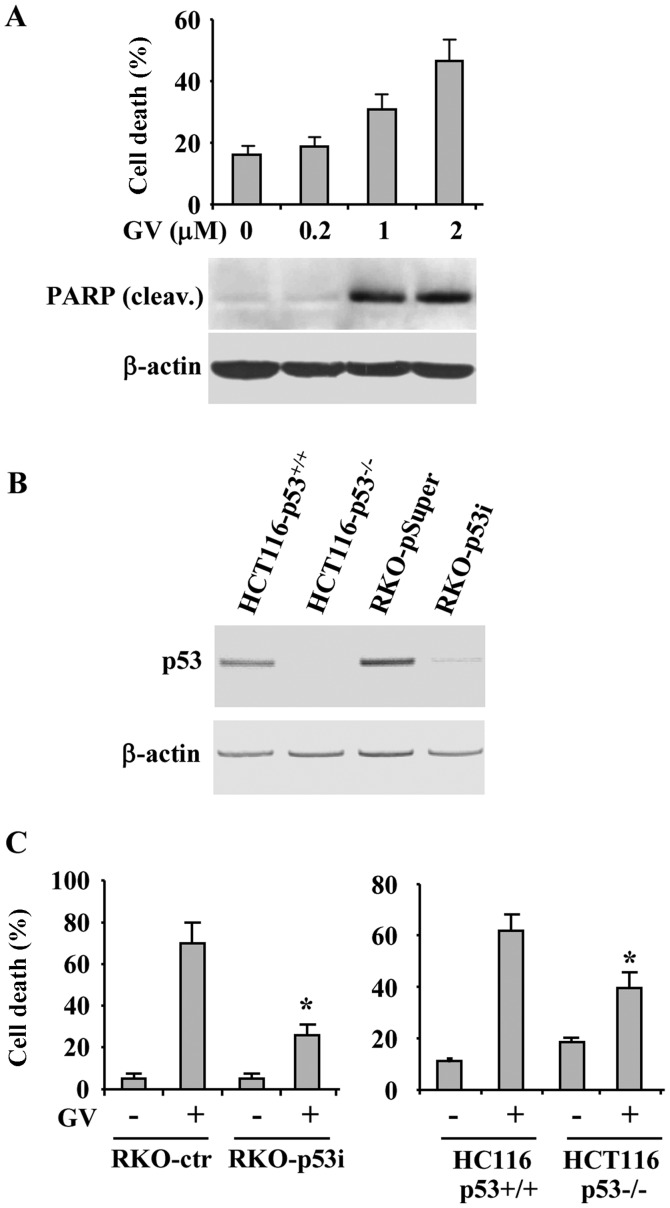
Gentian violet (GV)-induced cell death is impaired by p53 inhibition. (A) ADF cells (2×10^5^) were plated at subconfluence in 60-mm Petri dish and the day after treated with GV (0.2–1–2 *μ*M) for 24 h. Cell death was measured by trypan blue exclusion assay and expressed as percentage ± SD of two independent experiments. In the lower panel is shown western immunoblotting performed on equal amount of total cell extracts to detect PARP cleavage level. Anti-β-actin was used as protein loading control. (B) Semi-quantitative RT-PCR analyses of p53 expression in HCT116-p53^+/+^ and -p53^−/−^ cells and in RKO-Ctr and -sip53 cells. β-actin was used as internal control. (C) Cell death analyses of the indicated cells were (2×10^5^) at subconfluence in 60-mm Petri dish and the day after treated with GV (1 *μ*M) for 24 h. Cell death was measured by trypan blue exclusion assay and expressed as percentage ± SD of two independent experiments. ^*^P<0.001.

Finally, the GV effect on wtp53-carrying cancer cell death was tested by viability assay. Increasing concentration of GV progressively augmented cancer cell death with concomitant PARP cleavage (Fig. 4A), a marker of apoptotic cell death. To evaluate the role played by p53 in GV-induced cancer cell death we took advantage of a cell line stably interfered for p53 function (RKO-p53i) (18) and of HCT116-p53^−/−^ cells (Fig. 4B). RKO and HCT116 control and p53 negative cells were treated with GV and 24 h later cell viability was measured by trypan blue exclusion. As reported in Fig. 4C, cells deprived of p53 function showed reduced sensitivity to GV, compared to wtp53-carrying cells, with significant reduction of cell death, suggesting that the GV-induced cell death depends in part by p53 activation.

## Discussion

In this study we aimed at evaluating whether GV-induced cancer cell death could depend on p53 activation. GV was shown to induce the DNA binding and transactivation activities of p53 in the human colon cancer cells RKO and HCT116 and in glioblastoma ADF cells. The GV-induced p53 activation correlated with cancer cell death. Thus, this biological outcome was reduced in wtp53-negative cells as compared to wtp53-carrying cells. This implies that the GV anticancer activity was in part dependent on wtp53 activation.

GV has a long history of human use as anti-bacterial, antimycotic and antiparasitic agent. More recently, GV has been used also in different research fields (10) and has been shown to have strong anticancer activities in mice and human without having mutagen effect in humans (11–13). Thus, GV has been shown to be safe for human treatment (25). GV may have strong inhibitory activity against NADPH oxidases (Nox genes) (15). Among Nox genes, Nox1 has been reported to generate reactive oxygen (ROS) which in turn triggers the angiogenic switch (7), a hallmark of rapidly growing solid tumors (26). Moreover, we previously showed that Nox1 inhibits p53 transcriptional activity and oncosuppressor function by impairing p53 acetylation (6). Herein we found that GV was able to overcome the Nox1-induced p53 inhibition and restore p53 transcriptional activity. Nox1 is often overexpressed in tumors (27,28) and induces genome instability (29) which may account for p53 inhibition even in the absence of p53 mutation (30). Inactivation of p53 function by gene mutation or deregulation of wild-type p53 protein are common in human cancers and are indeed associated with increased cancer resistance to chemo- and radiotherapy (30). That is why significant efforts towards reactivation of defective p53 are underway, because functional p53 is considered a key factor for efficient antitumor drug response and apoptotic clearance of cancer cells (31). In this respect, a molecule such as GV that inhibits Nox1 activity may be an interesting agent to restore p53 function for anticancer activity.

More interestingly, though, we found that GV could directly induce p53/DNA binding and transactivation activities. Although GV stimulated the transactivation function of p53, the precise mechanism responsible for this activation needs to be determined. Established pathways of p53 activation that involve increased p53 stability or enhanced site-specific DNA binding (32–34) appeared to play a role in the p53 activation response to GV-treatment. The stabilization of p53 could depend on GV-induced DNA damage, as evidenced by H2AX phosphorylation in the position of Ser139 producing γH2AX that in general occurs in response to formation of double strand brakes (DSB) and is an early sign of replication stalling (24). Thus, the oncosuppressor p53 is activated in response to a variety of cellular stress signals, including DNA damage (1). However, different mechanisms cannot be excluded and need further evaluation.

In previous studies we reported that ADF glioblastoma cells carried an endogenous wild-type p53 whose transcriptional activity could not be activated by chemotherapeutic agent such as ADR, although the molecular mechanisms of such inhibition are still elusive (19,20). In the present study, GV was able to activate endogenous p53 in ADF cells, suggesting that different mechanisms other than DNA damage might be responsible of p53 activation in this cell line. In summary, our results indicate that while p53 induction by GV appears to be mediated to a large extent by increased p53 stability, it is unclear whether GV can have additional direct or indirect effects that induce p53 transactivation activity, such as protein phosphorylation and acetylation that may modulate the binding of p53 binding to DNA (35). Future experiments will be required to clarify this issue.

The role of p53 in GV-induced antitumor activity was evaluated by using cells with lack of (HCT116-p53^−/−^) or reduced p53 activity (RKO-sip53). GV was able to efficiently induce apoptotic cell death in wtp53-carrying cells and such biological outcome was reduced in cells deprived of p53 function.

In conclusion, the present study, shows for the first time the effect of GV on p53 activation, suggesting that the GV-mediated anticancer effect may in part depend on induction of the DNA binding and transactivation functions of p53. Future *in vitro* and *in vivo* studies will help to give more insight into unveiling the molecular mechanisms of GV activity and the potential role of GV alone or in combination with chemotherapeutic agents in cancer therapy.
